# [^18^F]AlF-NOTA-FAPI-04 PET/CT uptake in metastatic lesions on PET/CT imaging might distinguish different pathological types of lung cancer

**DOI:** 10.1007/s00259-021-05638-z

**Published:** 2021-12-06

**Authors:** Yuchun Wei, Kai Cheng, Zheng Fu, Jinsong Zheng, Zhengshuai Mu, Chenglong Zhao, Xiaoli Liu, Shijie Wang, Jinming Yu, Shuanghu Yuan

**Affiliations:** 1 Cheeloo College of Medicine, Shandong University, Jinan, China; 2 Department of Radiology, Shandong Cancer Hospital and Institute, Shandong First Medical University, Shandong Academy of Medical Sciences, Jinan, 250117 Shandong China; 3 Department of PET/CT Center, Shandong Cancer Hospital and Institute, Shandong First Medical University, Shandong Academy of Medical Sciences, Jinan, Shandong China; 4 Department of Pathology, Shandong Cancer Hospital and Institute, Shandong First Medical University, Shandong Academy of Medical Sciences, Jinan, Shandong China; 5 Shandong Provincial Key Laboratory of Radiation Oncology, Shandong Cancer Hospital and Institute, Shandong First Medical University, Shandong Academy of Medical Sciences, Jinan, Shandong Province, China

**Keywords:** Fibroblast activation protein, FAPI, PET/CT, Lung cancer

## Abstract

**Purpose:**

Heterogeneity is found in the tumor microenvironment among different pathological types of tumors. Radionuclide-labeled fibroblast-activation-protein inhibitor (FAPI), as an important tracer for non-invasive imaging of the tumor microenvironment, can be used to evaluate the expression of FAP in cancer-associated fibroblasts, macrophages, and tumor cells. Our aim was to explore the ability of [^18^F]AlF-NOTA-FAPI-04 positron emission tomography (PET)/computed tomography (CT) to distinguish different types of lung cancer by evaluating the uptake of this tracer in primary and metastatic lesions.

**Methods:**

We prospectively enrolled 61 patients with histopathologically proven primary lung cancer with metastases. PET/CT scanning was performed before any antitumor therapy and 1 h after injection of 235.10 ± 3.89 MBq of [^18^F]AlF-NOTA-FAPI-04. Maximum standard uptake values (SUV_max_) were calculated for comparison among primary and metastatic lesions. Immunohistochemical staining for FAP was performed on tumor specimens.

**Results:**

Sixty-one patients with adenocarcinoma (ADC, *n* = 30), squamous cell carcinoma (SCC, *n* = 17), and small cell lung cancer (SCLC, *n* = 14) were enrolled in this study, and 61 primary tumors and 199 metastases were evaluated. No difference in [^18^F]AlF-NOTA-FAPI-04 uptake was observed among primary ADC, SCC, and SCLC tumors (*P* = 0.198). Additionally, no difference in uptake was found between primary and metastatic lesions of lung cancer with the same pathological type (*P* > 0.05). However, uptake did differ among metastases of differing pathological types (*P* < 0.001). The SUV_max_ of metastatic lymph nodes was highest for SCC, followed by ADC and then SCLC (*P* < 0.001). The SUV_max_ of bone metastases also was highest for SCC, followed by ADC and SCLC (*P* < 0.05), but no difference was observed between ADC and SCLC. The SUV_max_ of metastases in other organs was higher in SCC cases than in ADC cases but did not differ between SCC and SCLC or ADC and SCLC. Bone metastases exhibited higher uptake than those of lymph nodes and other organs in SCC and ADC (*P* < 0.05) but not in SCLC. Positive correlations were found between FAPI uptake and FAP expression in surgical plus biopsy specimens (*r* = 0.439, *P* = 0.012) and surgical specimens (*r* = 0.938, *P* = 0.005).

**Conclusion:**

[^18^F]AlF-NOTA-FAPI-04 PET/CT imaging revealed differences in FAP expression in metastases of lung cancer, with the highest expression specifically in bone metastases, and thus, may be valuable for distinguishing different pathological types of lung cancer.

**Supplementary Information:**

The online version contains supplementary material available at 10.1007/s00259-021-05638-z.

## Introduction

Lung cancer is the most commonly diagnosed and deadliest cancer worldwide. About 40% of cases are diagnosed at an advanced stage, and the 5-year survival rate of patients with advanced lung cancer is less than 10% [1, 2]. Given the high mortality rate associated with lung cancer, a comprehensive understanding of the key biological characteristics closely related to the efficacy of lung cancer treatments is of great significance to improving outcomes among lung cancer patients.

Fibroblast activation protein (FAP) is a membrane-anchored peptidase that belongs to the dipeptidyl peptidase-4 (DPP-4) families and has dipeptidyl peptidase and endopeptidase activities [3, 4]. FAP expression has been reported on the surface of tumor stromal cells, macrophages, and tumor cells [5]. It is highly expressed in more than 90% of epithelial tumors [6] but expressed at low levels in physiological conditions [7, 8]. For this reason, it is often used as a marker of pro-tumorigenic stroma [9]. In healthy tissues, the stroma mainly acts as a barrier against tumor formation. However, the presence of tumor cells can transform the stroma into an environment that is conducive to tumor growth [10, 11]. The coevolution and continuous participation of the stroma and tumor cells in the process of tumorigenesis are the main reasons for the heterogeneity of the tumor microenvironment.

FAPI can specifically bind with FAP, and thus, its use in PET/CT imaging can allow the detection of FAP in the tumor microenvironment, providing insight into the biological characteristics of tumors. In this study, we quantified the tumor uptake of [^18^F]AlF-NOTA-FAPI-04 on PET/CT for different pathological types of lung cancer and metastatic tumors to characterize FAP expression in lung cancer and to explore the ability of [^18^F]AlF-NOTA-FAPI-04 PET/CT to distinguish different types of lung cancer.

## Materials and methods

### Patients

Patients were enrolled according to the following criteria: ① histologically confirmed lung cancer; ② age ≥ 18 years; ③ pathological subtypes of adenocarcinoma (ADC), squamous cell carcinoma (SCC), or small cell lung cancer (SCLC); ④ Eastern Cooperative Oncology Group performance status score of 0–2; and ⑤ voluntary participation in the study.

The following exclusion criteria were applied: ① special types of tumors, such as large cell lung cancer, adenosquamous carcinoma, and small salivary gland cell cancer; ② administration of any antitumor therapy before the [^18^F]AlF-NOTA-FAPI-04 PET/CT scan; and ③ recurrence or progression.

**Fig. 1:**
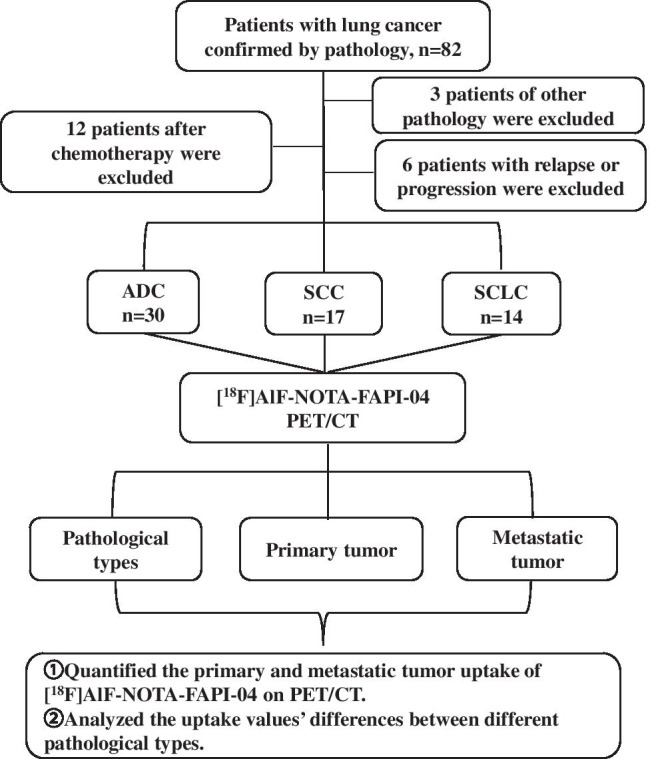
Research flowchart

A flow chart of the study is shown in Fig. 1. Sixty-one lung cancer patients with ADC (30/61), SCC (17/61), or SCLC (14/61) were randomly recruited from December 2020 to June 2021 and included in the final analysis. Our investigation was performed under the conditions of the updated Declaration of Helsinki (unproven interventions in clinical practice). This prospective study was approved by the local ethics committee of Shandong Cancer Hospital and Institute, and each patient provided written and informed consent for inclusion in the study.

### Compound synthesis and radiochemistry

NOTA-FAPI-04 was radiolabeled with ^18^F according to the following procedure: 2–10 GBq ^18^F fluoride produced by medical cyclotron (MINITRACE Cyclotron, GE, USA) was trapped on an anion exchange cartridge (Waters Plus QMA Light Cartridge, preconditioned with 5 mL saline and 10 mL water) and eluted with 0.30 mL saline. A solution of NOTA-FAPI-04 (100 μg, 130 nmol) in sodium acetate buffer (60 μL, pH 4, 0.1 M) was added to a solution of AlCl_3_ (6 μL, 10 mM in sodium acetate buffer, pH 4, 0.1 M) and 200 µL acetonitrile (≥ 99.9% Sigma-Aldrich, USA). ^18^F-fluoride (700 ~ 3300 MBq) in 100 µL target water was then added to the reaction solution sequentially. This mixture was heated for 10 min at 110 ℃. After dilution with water (10 mL), the reaction solution was transferred to a C18 Cartridge (Waters C18 Plus Light Cartridge, preconditioned with 10 mL ethanol and 10 mL water). The cartridge was washed with 10 mL water again, and then the desired product was eluted with 0.6 mL ethanol. The product was reconstituted in saline and passed through a 0.22-μm Millipore filter into a sterile vial.

**Fig. 2:**
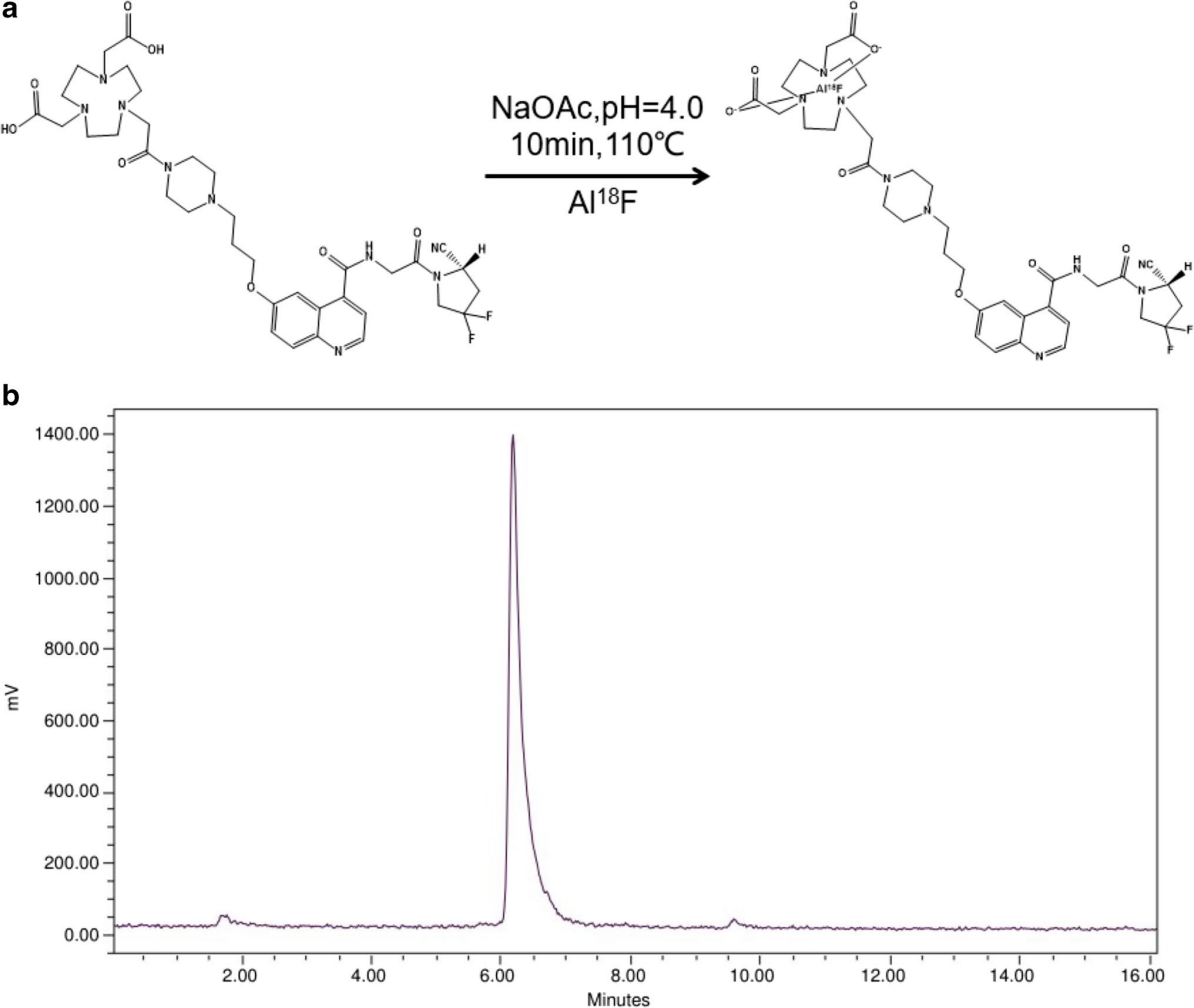
Chemical structural formula of [^18^F]AlF-NOTA-FAPI-04 (**a**). Radioactivity high-performance liquid chromatography (HPLC) of [^18^F]AlF-NOTA-FAPI-04 (**b**). The radiochemical purity of the final product measured by HPLC was > 98% in 6.3 min, and the specific activity was approximately 20 GBq/μmol

For quality control purposes, a portion of the product was diluted and injected onto an analytical C18 HPLC column for assessment of radiochemical purity. The retention time for [^18^F]AlF-NOTA-FAPI-04 was about 6.3 min. The radiochemical purity of [^18^F]AlF-NOTA-FAPI-04 exceeded 98%, and its specific radioactivity exceeded 20 GBq/μmol (Fig. 2).

### [^18^F]AlF-NOTA-FAPI-04 PET/CT scanning

Fasting and blood glucose measurements were not required or requested before imaging examinations. Patients received an intravenous injection of 4.81 MBq/kg (0.12 mCi/kg) [^18^F]AlF-NOTA-FAPI-04 and then rested for approximately 60 min. Scanning was then performed with an integrated in-line PET/CT system (GEMINI TF Big Bore; Philips Healthcare, Cleveland, OH, USA). Whole-body CT scans were acquired for attenuation correction using a low-dose protocol (300 mAs, 120 kV, a 512 × 512 matrix, rotation time of 1.0 s, and pitch index of 0.688; reconstructed with a soft-tissue kernel to a slice thickness of 2 mm). Subsequently, PET data were acquired in 3-dimensional mode with a 200 × 200 matrix with 1-min imaging time per bed position. After randoms, decay, and scatter correction, the data were reconstructed (Body-ctac-SB. Lstcln, Biograph 3D iterative reconstruction software, TOF correction). The patients continued normal shallow respiration during image acquisition.

### Imaging analysis

The images were attenuation-corrected with the transmission data from CT. The attenuation-corrected PET images, CT images, and fused PET/CT images, which were displayed as coronal, sagittal, and transaxial slices, were viewed and analyzed on a Nuclear Medicine Information System (Beijing Mozi Healthcare Ltd., Beijing, China). Two experienced, board-certified nuclear medicine physicians (Fu Z and Zheng J) visually assessed the [^18^F]AlF-NOTA-FAPI-04 PET/CT images and achieved consensus regarding the image interpretations for primary and metastatic tumors. The sites and the intensity of uptake in the tumors were recorded. Increased radioactivity compared with the background uptake and anatomical imaging (CT and MRI, et al.) findings in accordance with the characteristics of tumors were defined as being positive. Benign lesions such as sites of arthritis, rheumatic diseases, and old scars were not evaluated. For controversial tumor lesions, the two physicians discussed the findings while also considering other imaging examinations such as MRI or enhanced CT, and an agreement was reached.

Tumor tracer uptake was quantified by the same nuclear medicine physicians (Fu Z and Zheng J) according to the maximum standard uptake value (SUV_max_) at 1 h after injection. For calculation of the SUV, circular regions of interest (ROIs) were drawn around the tumor lesions with a focally increased uptake in transaxial slices and automatically adapted to a 3-dimensional volume with a 30% isocontour.

### Immunohistochemistry

Immunohistochemical staining was performed to confirm FAP expression in the tumor tissues. Tissues obtained by biopsy were formalin-fixed and paraffin-embedded before being cut into 3- to 5-µm-thick sections on a macrotome (Microm HM 450; GMI, Ramsey, MN, USA). The sections were then immunostained with a FAP-alpha antibody (1:100 dilution, ab53066; Abcam, Cambridge, MA, USA). A total of 32 specimens, including 26 biopsy specimens and 6 surgical specimens, were subjected to immunohistochemical staining and result analysis. FAP-positive cells were stained with brown-yellow granules or masses appearing in both the cell membrane and the cytoplasm. Both the intensity and percentage of positive cells were measured. The staining intensity was graded according to the following four classes: 0 = undetectable; 1 = faint buff; 2 = moderate buff; and 3 = high buff or sepia. FAP-positive cell percentage analysis and image acquisition were performed using the Mantra multispectral imaging platform (Vectra 3, PerkinElmer). Areas with nontumor or residual normal tissue were excluded from the analysis. Representative regions of interest were chosen by the pathologist, and multiple fields of view were acquired at × 20 resolution as multispectral images. Cell identification was performed as described previously [12], using supervised machine learning algorithms within inForm 2.3 (PerkinElmer). Thresholds for “positive” staining and the accuracy of phenotypic algorithms were optimized and confirmed by the pathologist (Zhao C) for each case. Using the percentage of stained cells × staining intensity, the integrated scoring was assessed. Cell expression was stratified as follows: 0 (negative) for no immunoreactivity, 1 for ≤ 25% positive cells, 2 for 25 to ≤ 50% positive cells, 3 for 50 to ≤ 75% positive cells, and 4 for > 75% positive cells. The product of the staining intensity and positive cell scores determined the final result for each section.

### Statistical analysis

The normally distributed measurement data were expressed as the mean ± SD, and non-normal measurement data were expressed as the median (25th to 75th percentiles). Comparisons between the two groups of normally distributed data were analyzed using a two-sample *t* test, and comparisons between the two groups of non-normal data were analyzed using the Mann–Whitney *U* test. Logistic regression analysis was used to analyze associations between SUVs and the FAP expression level. A *P* value less than 0.05 was considered statistically significant. All analyses were performed using Prism 9.1.2 (GraphPad, San Diego, CA, USA).

## Results

From December 2020 to June 2021, 61 patients diagnosed with lung cancer based on histological examinations at Shandong Cancer Hospital and Institute were enrolled in this study. A total of 61 primary tumors and 199 metastatic lesions were evaluated. The patients’ characteristics are presented in Table 1 (details are in Supplementary Table 1).

**Table 1:** Characteristics of the lung cancer patients (*n* = 61)

Age, mean ± SE (range)	61.52 ± 9.05 (37–83)
Male, *n* (%)	45 (73.77%)
Female, *n* (%)	16 (26.23%)
Pathological type, *n* (%)	
ADC	30 (49.18%)
SCC	17 (27.87%)
SCLC	14 (22.95%)
Primary tumors, *n*	61
Metastases, *n*	199

*SE*, standard error; *SCC*, squamous cell carcinoma; *ADC*, adenocarcinoma; *SCLC*, small cell lung carcinoma

### [^18^F]AlF-NOTA-FAPI-04 uptake in primary tumors of differing pathological type and comparison of uptake between primary and metastatic lesions

**Fig. 3:**
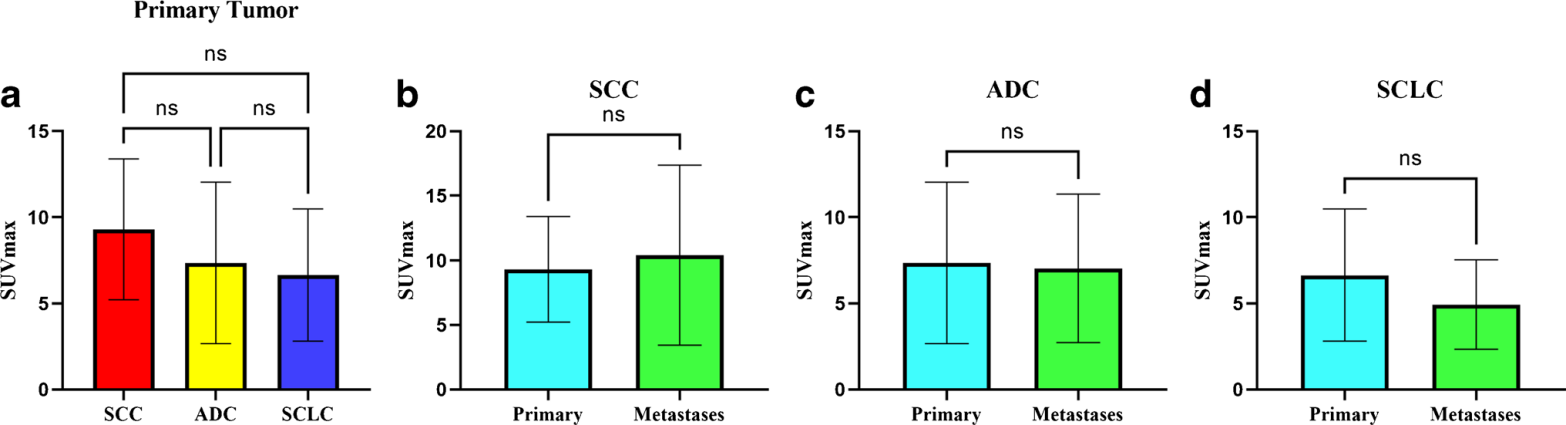
[^18^F]AlF-NOTA-FAPI-04 uptake in primary tumors and metastases. *ns*, not significant

**Fig. 4:**
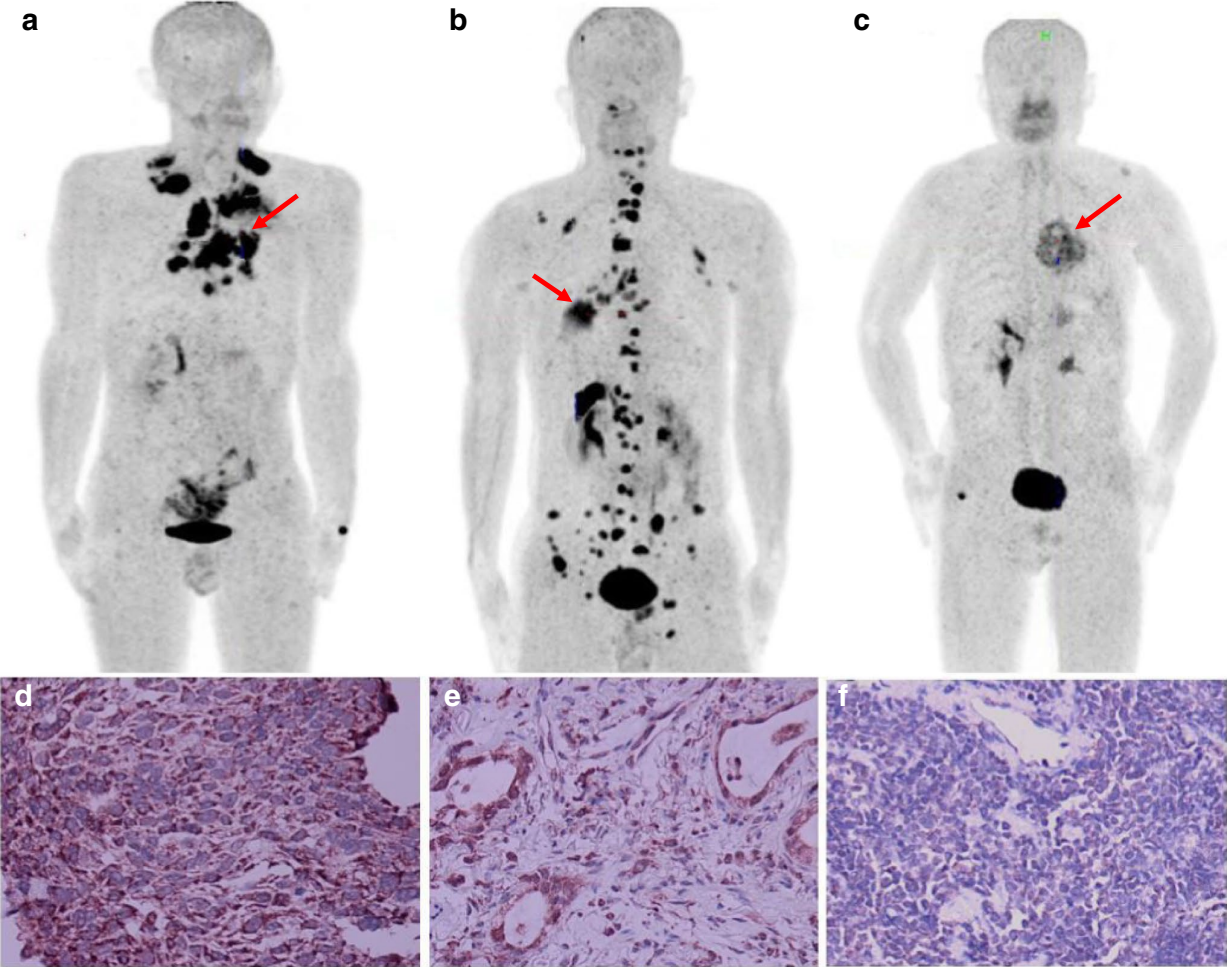
Representative [^18^F]AlF-NOTA-FAPI-04 PET/CT scans from patients with SCC (**a** SUV_max_ = 13.64), ADC (**b** SUV_max_ = 6.88) and SCLC (**c** SUV_max_ = 4.60). **d** Strong FAP staining of primary ADC tumor (× 200). **e** Moderate FAP staining of primary SCC tumor (× 200). **d** Weak FAP staining of primary SCLC tumor (× 200). Primary tumors are indicated by red arrows

The SUV_max_ of [^18^F]AlF-NOTA-FAPI-04 in primary tumors did not differ significantly among SCC 9.51 (5.18 ~ 12.63), ADC (7.36 ± 4.69), and SCLC (6.65 ± 3.83) tumors (*P* > 0.05; Table 2 and Fig. 3a). Additionally, [^18^F]AlF-NOTA-FAPI-04 uptake values in primary and metastatic lesions were comparable in patients with the same pathological type of lung cancer (Fig. 3b, c, d, and Fig. 4).

**Table 2:** SUV_max_ of primary tumors and metastases of different pathological types

	ADC	SCC	SCLC	*P*
Primary tumors	7.36 ± 4.69 (*n* = 30)	9.51 (5.18 ~ 12.63) (*n* = 17)	6.65 ± 3.83 (*n* = 14)	0.113
Metastases	7.03 ± 4.30 (*n* = 137)	10.41 ± 6.96 (*n* = 25)	4.94 ± 2.60 (*n* = 37)	< 0.001
Lymph node	6.61 ± 3.58 (*n* = 63)	9.05 (6.88 ~ 10.12) (*n* = 16)	4.41 ± 3.57 (*n* = 19)	< 0.001
Bone	9.14 ± 8.25 (*n* = 44)	26.27 (2.50 ~ *N*) (*n* = 3)	5.52 ± 4.07 (*n* = 12)	0.019
Liver	3.50 (2.78 ~ *N*) (*n* = 3)	3.38 (2.89 ~ *N*) (*n* = 2)	4.79 (3.46 ~ *N*) (*n* = 3)	0.211
Brain	2.85 (2.32 ~ 4.17) (*n* = 7)	17.66 (*n* = 1)	5.16 (*n* = 1)	
Adrenal gland	6.24 ± 4.45 (*n* = 6)	11.78 (*n* = 1)	/	
Pleura	3.88 (2.32 ~ 9.57) (*n* = 7)	8.95 (3.68 ~ *N*) (*n* = 2)	6.79 (*n* = 1)	
Peritoneum	6.99 (5.18 ~ 8.19) (*n* = 4)	/	/	
Intrapulmonary	1.71 (0.97 ~ *N*) (*n* = 2)	/	5.68 (*n* = 1)	
Soft tissue	6.55 (*n* = 1)	/	/	

*SCC*, squamous cell carcinoma; *ADC*, adenocarcinoma; *SCLC*, small cell lung carcinoma; *N*, not mentioned

### Comparison of [^18^F]AlF-NOTA-FAPI-04uptake between metastases of the same organs in patients with different pathological types of lung cancer

**Fig. 5:**
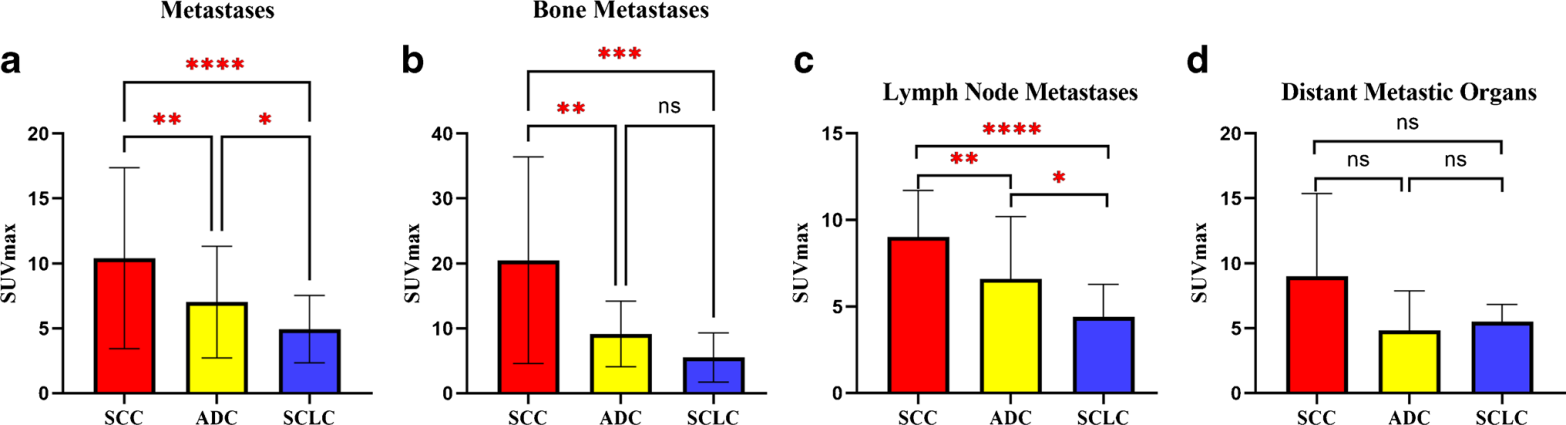
Differences in uptake values between metastases among different pathological types of lung cancer. *p < 0.05; **p < 0.01; ***p < 0.001; ****p < 0.0001

A significant difference in the SUV_max_ of [^18^F]AlF-NOTA-FAPI-04 was observed among metastases of different pathological types of lung cancer (*P* < 0.001; Fig. 5a). Metastases from SCC showed the highest SUV_max_ (10.41 ± 6.96), followed by ADC (7.03 ± 4.30) and then SCLC (4.94 ± 2.60). The differences between each pair of pathological types were all significant, with *P* values of 0.0019, < 0.0001, and 0.033 for the comparisons of SCC vs. ADC, SCC vs. SCLC, and ADC vs. SCLC, respectively.

For bone metastases (Fig. 5b), the SUV_max_ of metastases from SCC 26.27 (2.50 ~ *N*) was significantly higher than the SUV_max_ of metastases from ADC (9.14 ± 8.25; *P* = 0.0036) or SCLC (5.52 ± 4.07; *P* = 0.0004). No significant difference was observed between the uptake values in bone metastases from ADC and SCLC (*P* = 0.1273).

For lymph node metastases (Fig. 5c), the SUV_max_ of metastatic lymph nodes from SCC 9.05 (6.88 ~ 10.12) was higher than that of metastatic lymph nodes from ADC (6.61 ± 3.58; *P* = 0.0014) or SCLC (4.41 ± 3.57; *P* < 0.001). Additionally, the SUV_max_ of metastatic lymph nodes was higher for ADC than SCLC (*P* = 0.0256).

For metastases in other organs, including the brain, lung, adrenal gland, liver, pleura, peritoneum, and soft tissue (Fig. 5d), the SUV_max_ in metastatic lesions from SCC, ADC, and SCLC were 7.83 (3.48 ~ 15.09), 4.25 (2.33 ~ 6.49), and 5.42 (4.46 ~ 6.87), respectively. The differences between each pair of pathological types were not significant, with *P* values of 0.132, 0.749, and 0.252 for the comparisons of SCC vs. ADC, SCC vs. SCLC, and ADC vs. SCLC, respectively.

### Comparison of [^18^F]AlF-NOTA-FAPI-04 uptake between metastases of different organs in patients with the same pathological type of lung cancer

**Fig. 6:**
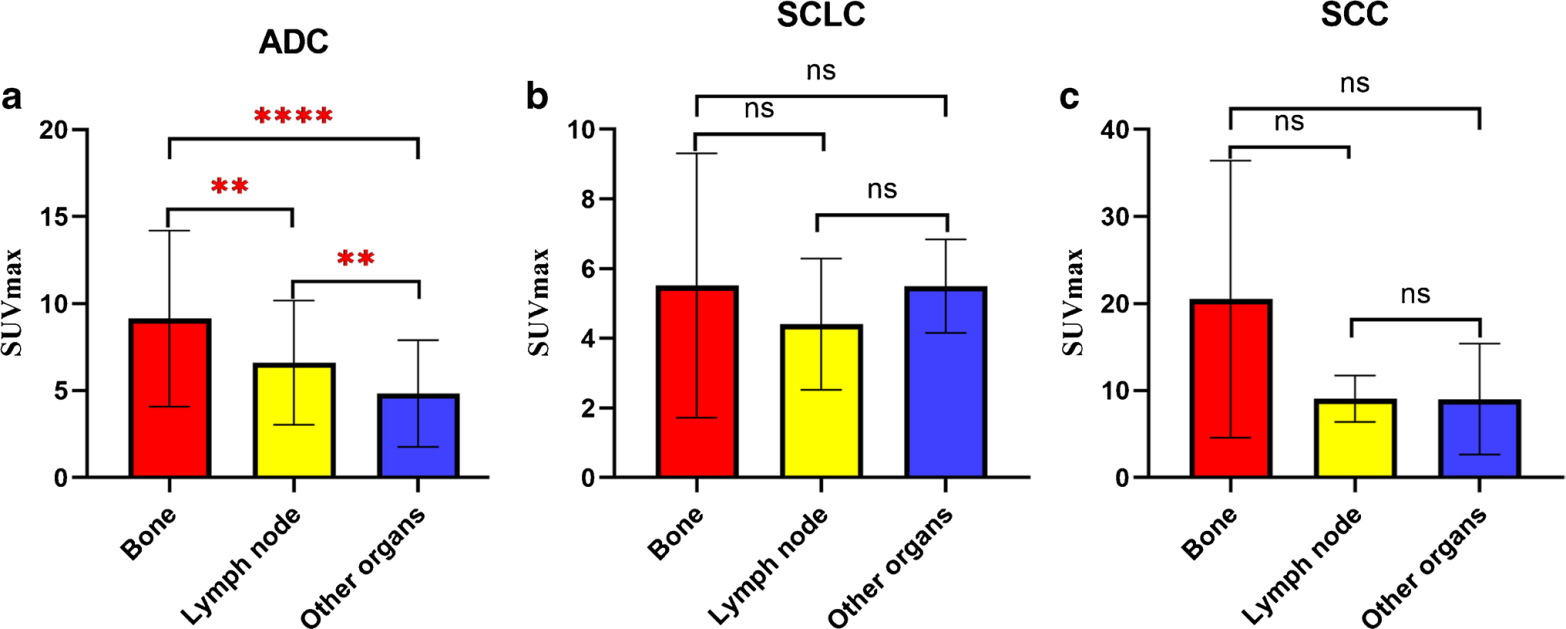
Differences in uptake values between different types of metastases. *ns*, not significant; *p < 0.05; **p < 0.01; ****p < 0.0001

**Fig. 7:**
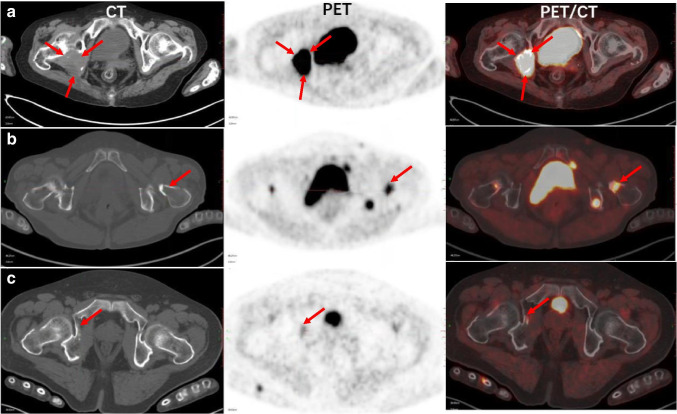
Representative [^18^F]AlF-NOTA-FAPI-04 PET/CT scans of bone metastasis in patients with SCC (**a** SUV_max_ = 26.27), ADC (**b** SUV_max_ = 13.2) and SCLC (**c** SUV_max_ = 6.24). Red arrow points to bone metastasis

In patients with ADC, the SUV_max_ for bone metastases, lymph node metastases, and metastases in other organs were 20.05 ± 9.19, 9.04 ± 0.67, and 7.83 (3.48 ~ 15.09), respectively (Fig. 6a and Fig. 7). The SUV_max_ in bone metastases was higher than the uptake values in lymph node metastases (*P* = 0.018) or metastases in other organs (*P* < 0.001), while the uptake values of lymph node metastases were higher than those of metastases in other organs (*P* = 0.008).

Different from the results obtained in ADC, the [^18^F]AlF-NOTA-FAPI-04 uptake values in bone metastases, metastatic lymph nodes, and metastases in other organs in patients with SCLC were 5.52 ± 1.10, 4.41 ± 0.43, and 5.42 (4.46 ~ 6.87), and those in patients with SCC were 26.27 (2.50 ~ *N*), 9.05 (6.88 ~ 10.12), and 7.83 (3.48 ~ 15.09), respectively, with no significant differences between them (all *P* > 0.05; Fig. 6b, c).

### Immunohistochemical validation and the correlation with [^18^F]AlF-NOTA-FAPI-04 uptake on PET

**Fig. 8:**
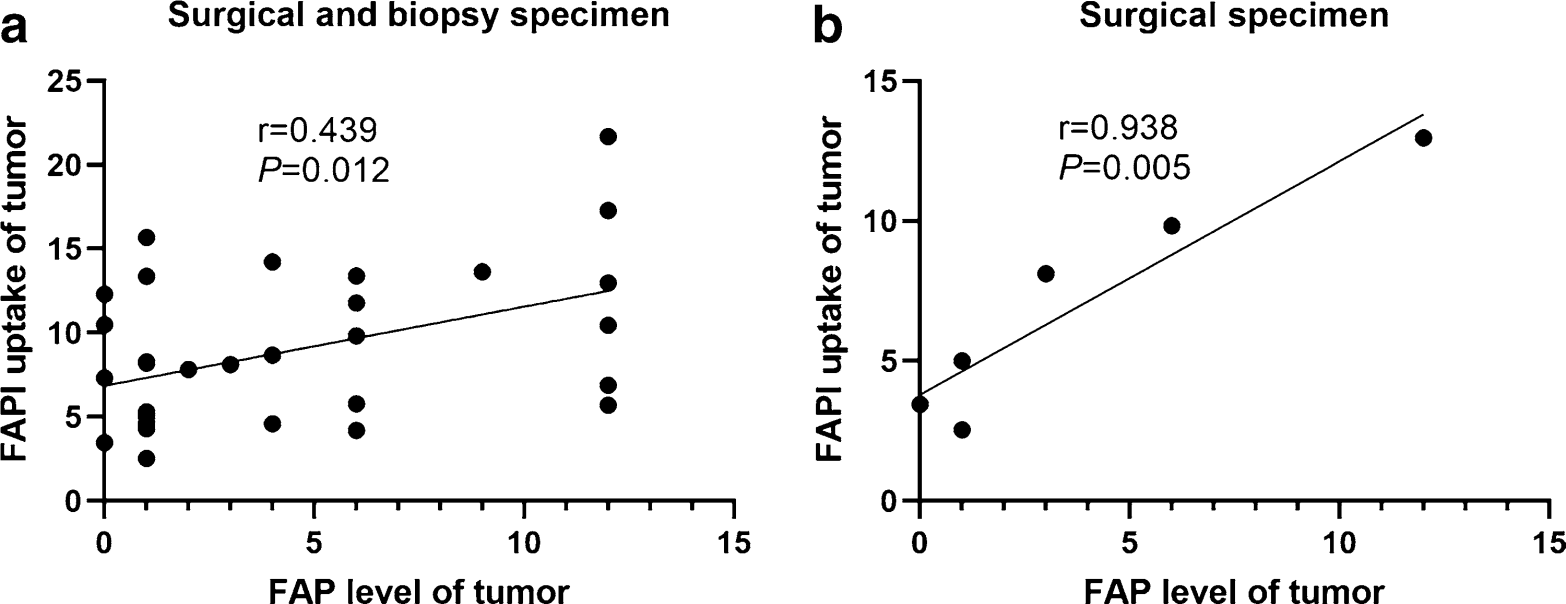
Correlation between FAPI uptake and ICH staining for FAP expression in tumors. Correlations were found between FAPI uptake and the FAP expression level in surgical and biopsy specimens (**a** *r* = 0.439, *P* = 0.012) and between FAPI uptake and the FAP expression level in surgical specimens (**b** *r* = 0.938, *P* = 0.005)

PET imaging was validated by IHC examination. Staining for FAP was found both in the cell membrane and the cytoplasm. The expression intensity of FAP in surgical plus biopsy specimens (*n* = 32) was 4.34 ± 0.77, and a moderate positive correlation was observed between FAPI uptake and the FAP expression level (*r* = 0.439, *P* = 0.012) (Fig. 8a). The expression intensity of FAP in surgical specimens (*n* = 6) was 3.83 ± 1.85, and a strong positive correlation was found between FAPI uptake and the FAP expression level (*r* = 0.938, *P* = 0.005) (Fig. 8b). A better correlation between FAPI uptake and FAP expression was observed in tumor surgical specimens than in surgical plus biopsy specimens, just as surgical specimens can show the true expression of FAP in tumors better than puncture specimens.

## Discussion

In this study, differences in [^18^F]AlF-NOTA-FAPI-04 uptake were observed among metastatic lung cancer lesions in different sites, which provide insights into the characteristics of FAP expression in the tumor microenvironment of lung cancer and can be helpful for distinguishing different pathological types of lung cancer via PET/CT imaging.

The results of this study showed no differences in [^18^F]AlF-NOTA-FAPI-04 uptake in primary lung tumors of different pathological types. To better characterize the natural expression of FAP in lung cancer, the lung cancer patients enrolled in this study had not yet received any antitumor therapy at the time of PET/CT imaging. The uptake values in primary tumors were lower than those reported previously for lung cancer (average SUV_max_ > 11) [13, 14], but the previous studies included some patients who had already received treatment. Thus, it remained unclear whether the uptake values reported in previous studies included metastatic tumors. No related studies on FAPI-04 uptake in patients with different pathological types of lung cancer were found in the literature prior to the present study.

Using FAP-specific PET imaging may allow noninvasive distinction of lung cancer pathological types between SCC, ADC, and SCLC. In this study, we found that the [^18^F]AlF-NOTA-FAPI-04 uptake values in metastases from SCC were the highest, followed by uptake values in metastases from ADC, with the lowest uptake values observed in metastases from SCLC. Lung cancer metastasis is a complex process that begins with the implantation of tumor cells and their interaction with stroma [10]. Then quiescent fibroblasts differentially respond to damage and become activated to support repair. These differentiated fibroblasts are called cancer-associated fibroblasts (CAFs) and can significantly promote tumorigenesis [15]. The process of tumor metastasis occurs in the context of considerable tumor heterogeneity. A previous study found that increased tracer uptake on FAP-specific imaging had the ability to distinguish isocitrate dehydrogenase (IDH)-wild-type glioblastomas and high-grade IDH-mutant astrocytoma [16]. In addition, [^68^ Ga]Ga-FAPI-04 uptake showed a high sensitivity for distinguishing poorly differentiated hepatic malignancies, with the results confirmed by pathology [17]. Therefore, FAP-specific imaging in tumors may be useful for follow-up studies.

Patients with advanced lung cancer have local and distant tissue and organ metastases. FAP is often highly expressed in the pathophysiological process of the desmoplastic response. SCLC exhibits poor differentiation, a high degree of malignancy and rapid development [18, 19]. Although it is typically diagnosed in an advanced stage, the expression levels of FAP in different metastatic lesions usually have not reached a significant level of variance, which may be the reason no significant difference in FAPI-04 uptake was observed among metastases from SCLC. The growth patterns of SCC and ADC originate in epithelial cells and mostly involve accumulative growth, often showing solid density [20–22]. Well-differentiated SCC is associated with a good prognosis [23, 24], and metastasis typically occurs over a long time, resulting in differential expression of FAP in metastases of different organs and tissues.

Bone metastasis is common in patients with advanced lung cancer, with the reported incidence of bone metastasis in non-small cell lung cancer ranging from 30 to 55% [25, 26]. In extensive small cell lung cancer, about 40% of patients have bone metastasis [27]. In the present study, patients with ADC and SCLC often had multiple systemic metastases, and FAPI-04 uptake in bone metastases was higher than that in other tissues and organs. At present, radiotherapy combined with opioid analgesia is commonly used in the clinical treatment of bone metastasis, but it can only partially or temporarily relieve the symptoms of metastatic lesions. FAPI-04 was reported to be a promising tracer for both imaging and improving the potency of targeted treatment in cancer patients [28]. [^68^ Ga]Ga-FAPI-04 PETCT scans present high absorbance in lesions associated with metastases of breast cancer, and pain symptoms were found to decrease with the use of a low dose of [^90^Y]Y-FAPI-04 [29]. Theranostic targeting of FAP in the tumor stroma provides a new therapeutic idea for the treatment of patients with systemic metastases including multiple bone metastases and metastases in other tissues and organs.

Due to the limitations of this study, such as the small number of patients and the resultant inability to compare the SUVs of some of metastases tumors for individual cancers, further studies are required to confirm the ability to distinguish pathological lung cancer types based on [^18^F]AlF-NOTA-FAPI-04 uptake values in metastases on PET/CT imaging.

## Conclusion

[^18^F]AlF-NOTA-FAPI-04 PET/CT imaging revealed differential expression of FAP in metastases of lung cancer, especially in bone metastases, with no differences in uptake values observed among primary tumors of different pathological types of lung cancer. Thus, PET/CT imaging with this tracer may be valuable in planning therapeutic regimens for patients with advanced lung cancer.

## Supplementary Information

Below is the link to the electronic supplementary material.

Supplementary file1 (DOCX 19 KB)

## Data Availability

The datasets used or analyzed during the current study are available from the corresponding author on reasonable request.

## Code availability

All software applications or custom code are available in the public repository.
