# Supplementary material for: [18F]AlF-NOTA-FAPI-04 PET/CT uptake in metastatic lesions on PET/CT imaging might distinguish different pathological types of lung cancer
Source: Eur J Nucl Med Mol Imaging. 2021 Dec 6;49(5):1671–81. doi: 10.1007/s00259-021-05638-z (PMC8940861; doi:10.1007/s00259-021-05638-z)
Supplement: Supplementary file 1 — Supplementary file1 (DOCX 19 KB) [file 259_2021_5638_MOESM1_ESM.docx]

| **Supplement Table 1 Clinical characteristics of the included patients** | | | | | | |
| --- | --- | --- | --- | --- | --- | --- |
| **No.** | **FAPI (MBq)** | **Sex** | **Age (yrs)** | **TNM stage** | **Pathological specimen** | **Pathology** |
| 1 | 170.2 | F | 73 | IA | Surgery: Left lung | ADC |
| 2 | 266.4 | M | 65 | IA | Surgery: Right lung | ADC |
| 3 | 177.6 | F | 49 | IV | Biopsy: Left lung | ADC |
| 4 | 229.4 | M | 73 | IIIA | Biopsy: Right lung | ADC |
| 5 | 255.3 | M | 60 | IVB | Biopsy: 10L lymph node | ADC |
| 6 | 225.7 | M | 48 | IV | Biopsy: Mediastinal lymph nodes | ADC |
| 7 | 251.6 | M | 53 | IVA | Biopsy: Left lung | ADC |
| 8 | 222 | F | 64 | IV | Biopsy: Right lung | ADC |
| 9 | 203.5 | F | 55 | IV | Biopsy: Right lung | ADC |
| 10 | 266.4 | M | 65 | III | Surgery: Left lung; region 5, 6, 7, 9, 10, and 11 lymph node | ADC |
| 11 | 222 | F | 58 | IV | Biopsy: Left lung | ADC |
| 12 | 259 | M | 66 | IV | Biopsy: Right lung | ADC |
| 13 | 266.4 | M | 50 | IV | Biopsy: Pleura | ADC |
| 14 | 281.2 | M | 70 | IIIB | Biopsy: Right lung | ADC |
| 15 | 225.7 | M | 51 | IV | Biopsy: Right lung | ADC |
| 16 | 185 | F | 69 | IV | Biopsy: Left lung | ADC |
| 17 | 177.6 | F | 71 | IV | Biopsy: Right lung | ADC |
| 18 | 255.3 | M | 55 | IVB | Biopsy: Mediastinal lymph nodes | ADC |
| 19 | 259 | M | 74 | IV | Biopsy: Left lung | ADC |
| 20 | 240.5 | F | 54 | IV | Biopsy: Mediastinal lymph nodes | ADC |
| 21 | 247.9 | M | 42 | IV | Biopsy: Right lung | ADC |
| 22 | 229.4 | M | 64 | IV | Biopsy: Left lung | ADC |
| 23 | 244.2 | M | 37 | IV | Biopsy: Right lung | ADC |
| 24 | 240.5 | M | 56 | IV | Biopsy: Left lung | ADC |
| 25 | 188.7 | F | 58 | IV | Biopsy: Right lung | ADC |
| 26 | 240.5 | F | 65 | IIIA | Surgery: Right lung; pleura; region 5, 6, 7, 10, and 11 lymph node | ADC |
| 27 | 203.5 | M | 61 | IV | Biopsy: Right lung | ADC |
| 28 | 188.7 | M | 69 | IVA | Biopsy: Right lung | ADC |
| 29 | 222 | F | 63 | IVB | Biopsy: Left lung | ADC |
| 30 | 240.5 | F | 65 | IB | Surgery: Right lung; pleura; region 2, 4, 7, 10, and 12 lymph node | ADC |
| 31 | 225.70 | M | 63 | IV | Biopsy: Left lung | SCLC |
| 32 | 251.60 | M | 79 | IV | Biopsy: Left lung | SCLC |
| 33 | 210.90 | M | 62 | IIIA | Biopsy: Left lung | SCLC |
| 34 | 255.30 | M | 64 | IIIA | Biopsy: Left lung | SCLC |
| 35 | 247.90 | M | 58 | IV | Biopsy: Mediastinal lymph nodes | SCLC |
| 36 | 277.50 | M | 55 | IIIB | Biopsy: Left lung | SCLC |
| 37 | 240.50 | M | 72 | IV | Biopsy: Right lung | SCLC |
| 38 | 236.80 | F | 64 | IVA | Biopsy: Right lung | SCLC |
| 39 | 296.00 | M | 45 | IIIA | Biopsy: Right lung | SCLC |
| 40 | 192.40 | M | 56 | III | Biopsy: Mediastinal lymph nodes | SCLC |
| 41 | 203.50 | M | 62 | IIIA | Biopsy: Right lung | SCLC |
| 42 | 251.60 | M | 50 | IV | Biopsy: Left lung | SCLC |
| 43 | 273.80 | M | 63 | IIIA | Biopsy: Left lung | SCLC |
| 44 | 244.20 | M | 70 | IV | Biopsy: Left lung; mediastinal lymph nodes | SCLC |
| 45 | 262.70 | M | 53 | IIB | Surgery: Left lung; pleura; region 4, 5,7, 10, 11, and 12 lymph node | SCC |
| 46 | 255.30 | M | 61 | IIIA | Surgery: Left lung; pleura; region 4, 5, 6, 7, 10, and 11 lymph node | SCC |
| 47 | 210.90 | M | 63 | III | Biopsy: Right lung; mediastinal lymph node | SCC |
| 48 | 266.40 | F | 50 | IVA | Biopsy: Right lung | SCC |
| 49 | 185.00 | F | 68 | IV | Biopsy: Right lung | SCC |
| 50 | 229.40 | M | 64 | IV | Biopsy: Right lung | SCC |
| 51 | 214.60 | F | 63 | IIIA | Biopsy: Right lung | SCC |
| 52 | 255.30 | M | 54 | IIIB | Biopsy: Right lung | SCC |
| 53 | 210.90 | M | 69 | IIIC | Biopsy: Right lung, region of 7 lymph node | SCC |
| 54 | 214.60 | M | 70 | III | Biopsy: Right lung | SCC |
| 55 | 218.30 | M | 56 | IV | Biopsy: Left lung | SCC |
| 56 | 255.30 | M | 63 | III | Biopsy: Left lung | SCC |
| 57 | 244.20 | M | 70 | IIA | Biopsy: Right lung | SCC |
| 58 | 218.30 | M | 67 | IV | Biopsy: Left lung | SCC |
| 59 | 314.50 | M | 56 | IIIA | Biopsy: Left lung | SCC |
| 60 | 225.70 | M | 77 | IV | Biopsy: Right parasternal soft tissue | SCC |
| 61 | 266.40 | M | 83 | IV | Biopsy: Liver | SCC |
| **SCC, squamous cell carcinoma; ADC, adenocarcinoma; SCLC, small cell lung carcinoma.** | | | | | | |
|  |  |  |  |  |  |  |
